# Therapeutic Targeting of Aristolochic Acid Induced Uremic Toxin Retention, SMAD 2/3 and JNK/ERK Pathways in Tubulointerstitial Fibrosis: Nephroprotective Role of Propolis in Chronic Kidney Disease

**DOI:** 10.3390/toxins12060364

**Published:** 2020-06-02

**Authors:** Jia-Feng Chang, Chih-Yu Hsieh, Kuo-Cheng Lu, Yue-Wen Chen, Shih-Shin Liang, Chih-Cheng Lin, Chi-Feng Hung, Jian-Chiun Liou, Mai-Szu Wu

**Affiliations:** 1 Division of Nephrology, Department of Internal Medicine, Shuang Ho Hospital, Taipei Medical University, New Taipei City 235, Taiwan; cjf6699@gmail.com; 2 Division of Nephrology, Department of Internal Medicine, En Chu Kong Hospital, New Taipei City 237, Taiwan; fish37435@hotmail.com; 3 Graduate Institute of Aerospace and Undersea Medicine, Academy of Medicine, National Defense Medical Center, Taipei 114, Taiwan; 4 Department of Nursing, Yuanpei University of Medical Technology, Hsinchu 300, Taiwan; 5 Renal Care Joint Foundation, New Taipei City 220, Taiwan; 6 Graduate Institution of Biomedical and Pharmaceutical Science, College of Medicine, Fu Jen Catholic University, New Taipei City 242, Taiwan; 7 School of Biomedical Engineering, Taipei Medical University, Taipei 110, Taiwan; 8 Department of Pathology, Tri-Service General Hospital, National Defense Medical Center, Taipei 114, Taiwan; 9 Division of Nephrology, Department of Medicine, Fu Jen Catholic University Hospital, New Taipei City 242, Taiwan; Kuochenglu@gmail.com; 10 Department of Nephrology, Taipei Tzu Chi Hospital, Buddhist Tzu Chi Medical Foundation, New Taipei City 231, Taiwan; 11 Department of Biotechnology and Animal Science, National Ilan University, Yilan 260, Taiwan; chenyw@niu.edu.tw; 12 Institute of Biomedical Science, National Sun Yat-Sen University, Kaohsiung 804, Taiwan; liang0615@kmu.edu.tw; 13 Department of Biotechnology, College of Life Science, Kaohsiung Medical University, Kaohsiung 807, Taiwan; 14 Department of Biotechnology and Pharmaceutical, Yuanpei University, Hsinchu 300, Taiwan; lcc@mail.ypu.edu.tw; 15 School of Medicine, Fu Jen Catholic University, New Taipei City 242, Taiwan; 054317@mail.fju.edu.tw; 16 Division of Nephrology, Department of Internal Medicine, School of Medicine, College of Medicine, Taipei Medical University, Taipei 110, Taiwan

**Keywords:** aristolochic acid, uremic toxins, propolis extract, tubulointerstitial fibrosis, transforming growth factor-β, chronic kidney disease

## Abstract

The nephrotoxicity of aristolochic acids (AAs), p-cresyl sulfate (PCS) and indoxyl sulfate (IS) were well-documented, culminating in tubulointerstitial fibrosis (TIF), advanced chronic kidney disease (CKD) and fatal urothelial cancer. Nonetheless, information regarding the attenuation of AAs-induced nephropathy (AAN) and uremic toxin retention is scarce. Propolis is a versatile natural product, exerting anti-oxidant, anti-cancer and anti-fibrotic properties. We aimed to evaluate nephroprotective effects of propolis extract (PE) in a murine model. AAN was developed to retain circulating PCS and IS using C57BL/6 mice, mimicking human CKD. The kidney sizes/masses, renal function indicators, plasma concentrations of PCS/IS, tissue expressions of TIF, α-SMA, collagen IaI, collagen IV and signaling pathways in transforming growth factor-β (TGF-β) family were analyzed among the control, PE, AAN, and AAN-PE groups. PE ameliorated AAN-induced renal atrophy, renal function deterioration, TIF, plasma retention of PCS and IS. PE also suppressed α-SMA expression and deposition of collagen IaI and IV in the fibrotic epithelial-mesenchymal transition. Notably, PE treatment in AAN model inhibited not only SMAD 2/3-dependent pathways but also SMAD-independent JNK/ERK activation in the signaling cascades of TGF-β family. Through disrupting fibrotic epithelial-mesenchymal transition and TGF-β signaling transduction pathways, PE improves TIF and thereby facilitates renal excretion of PCS and IS in AAN. In light of multi-faced toxicity of AAs, PE may be capable of developing a new potential drug to treat CKD patients exposed to AAs.

## 1. Introduction

Aristolochic acids (AAs) are well-known nephrotoxins, while information regarding the attenuation of AAs-induced toxicity is scarce. AAs primarily damage renal tubulointerstitium, culminating in profound tubulointerstitial fibrosis (TIF), end-stage kidney disease (ESKD) and fatal urothelial cancer [1]. TIF is the outcome of multiple forms of chronic kidney disease (CKD), which is commonly caused by diabetes, hypertension and nephrotoxins [2]. The irreversible renal fibrosis and sustained retention of uremic toxins from the dietary-protein intake play a pivotal role in CKD pathogenesis [3]. In parallel with a decline in renal function, a myriad of uremic retention solutes exhibit pro-oxidant, pro-inflammation and pro-fibrotic effects on renal injury, leading to a vicious cycle [4,5]. That is, an accumulation of uremic toxins following impaired renal clearance inhibits renal metabolic capacity and induces progressive TIF [6,7]. Emerging evidences indicate the protein-bound non-dialyzable uremic toxins such as *p*-cresyl sulfate (PCS) and indoxyl sulfate (IS) are intricately associated with oxidative injury and fibrogenesis in CKD and diverse organ systems [8,9]. Furthermore, TIF is reminiscent of epithelial mesenchymal transition (EMT), excess deposition of extracellular matrix (ECM) components, collagens, SMAD 2/3-dependent and SMAD-independent JNK/ERK pathways in transforming growth factor-β (TGF-β) family signaling [10,11]. Nonetheless, a comprehensive treatment to inhibit AAs-induced TIF signaling cascades and PCS/IS accumulation is still lacking.

Propolis, a natural product rich in prenylated flavonoids, has been reported to exert versatile biological activities in our prior research, including anti-inflammasome [12], anti-oxidant [12], anti-diabetes [13,14] and anti-cancer properties [15]. Our recent rodent model of metabolic endotoxemia with obesity demonstrated that elimination of reactive oxygen species (ROS) using antioxidant therapy ameliorates kidney fibrosis as well as inflammation [16]. Considering pro-oxidant and pro-fibrotic effects of uremic retention solutes on human organ systems, the natural antioxidant PE may serve as a potential breakthrough treatment for AAs-induced nephropathy (AAN). Despite previously documented implications, the therapeutic effect of PE on TIF and PCS/IS retention remains elusive. Thus, we developed an experimental mouse model to explore therapeutic targets in AAN. We hypothesized that PE treatment in AAN model attenuated not only SMAD 2/3-dependent pathways but also SMAD-independent JNK/ERK activation in the signaling cascades of TGF-β family, contributing to a reversal of TIF and uremic burden.

## 2. Results

### 2.1. Working Model of CKD Mouse with AAN and Identification of Renal Function and Uremic Cachexia

**Figure 1:**
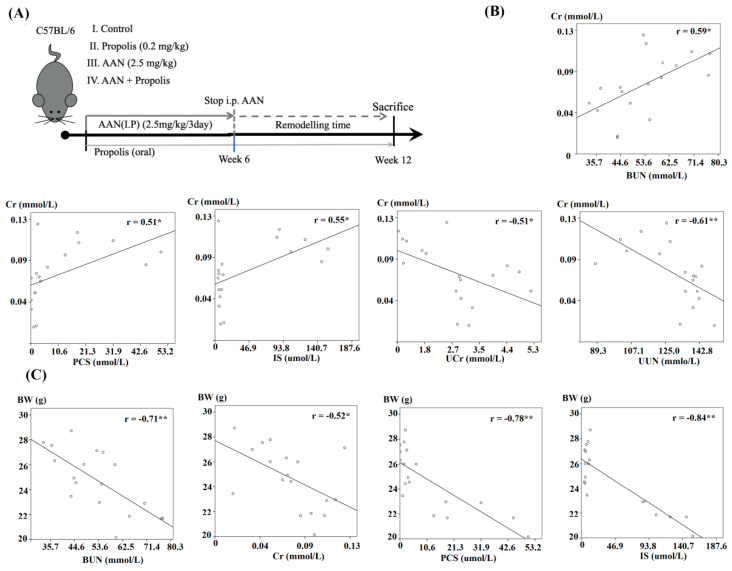
Working model of AAN mouse and identifying renal function decline and uremic cachexia. (**A**) Mice were randomized into four groups: Group I (control; IP injection of vehicle (DMSO) once every 3 days for 6 weeks and orally administered with vehicle (distilled water, 200 μL) everyday, 12 weeks; *n* = 6). Group II (PE alone; IP injection of vehicle and orally administered with PE (0.2 mg/kg in 200 μL vehicle), 12 weeks; *n* = 6). Group III (AAN; IP injection of AAI and orally administered with vehicle (200 μL) everyday, 12 weeks; *n* = 6), Group IV (PE + AAI treatment; IP injection of AAI and orally administered with PE, 12 weeks; *n* = 6). (**B**) Plasma concentration of Cr correlates with BUN, PCS, IS and impaired urinary excretion of waste products (Group I-III, *n* = 18). (**C**) BW negatively correlates with BUN, Cr and uremic toxins, indicative of uremic cachexia. Data are expressed as * *p* < 0.05 and ** *p* < 0.01 to compare the differences between the two indicated variables. AAI = aristolochic acid I; AAN = aristolochic acid nephropathy; BUN = blood urea nitrogen; BW = body weight; Cr = creatinine; IS = indoxyl sulfate; PCS = *p*-cresyl sulfate; UCr = urine creatinine; UUN = urine urea nitrogen.

To mimic the typical findings of advanced CKD in humans, we developed an experimental mouse model with AAN (Figure 1A). Under the circumstances of uremic inflammation, uremic cachexia highly prevalent among CKD patients is featured with anorexia, increased energy expenditure and decreased body weight (BW) and protein stores [17]. To identify the renal function of AAN mice, a series of correlation tests were analyzed. The scatter diagram illustrated positive correlations between plasma concentrations of BUN, PCS, IS and Cr were robust (Figure 1B), indicating that renal dysfunction led to the accumulation of various uremic toxins in the circulation. Plasma Cr negatively correlated with urine urea nitrogen (UUN) and urine creatinine (UCr), indicating that impaired urinary excretion of waste products due to CKD (Figure 1B). Our data revealed BW negatively correlated with BUN, Cr and uremic toxins, indicative of uremic cachexia (Figure 1C). Interestingly, there findings were compatible with previous studies that a significant inverse association between plasma uremic toxins and skeletal muscle mass in clinical observation and basic research [18].

### 2.2. AAN Mice Exhibit the Most Prominent Renal Atropy, TIF and Necrosis of Tubular Cells, Whereas PE Treatment Ameliorated Such Renal Damages

**Figure 2:**
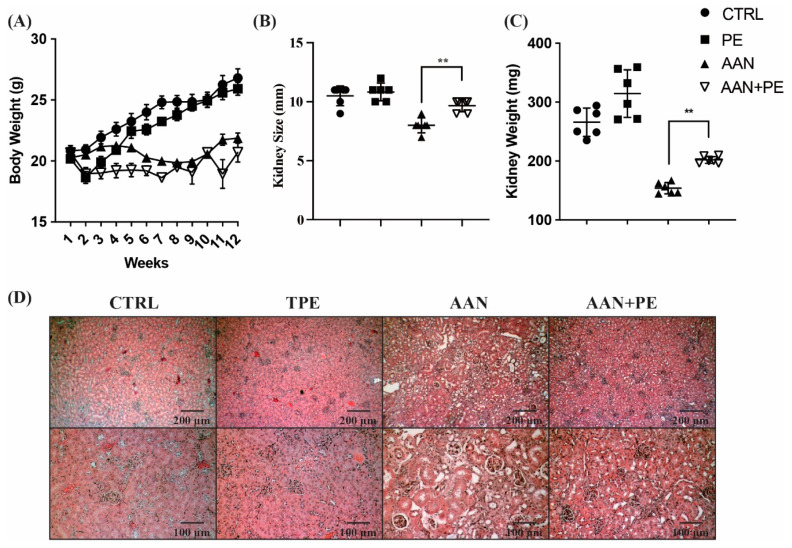
Comparisons of BW, kidney size and weight, and the histopathological evaluation of H&E stain among the control, PE, AAN and AAN-PE treatment groups. (**A**) BW of C57BL/6 mice with AAN treatment were lower than those without AAN. (**B**,**C**) AAN group without PE treatment exhibited the smallest kidney size and mass, and PE treatment ameliorated such renal atrophy. (**D**) H&E stain showed renal tubular cells in AAN group exhibited the most prominent cytoplasmic vacuolation, loss of cell-cell adhesion, apical-basal polarity and necrosis- irreversible cellular change with eventual sloughing and cell loss. *n* = 6 in each group; ** *p* < 0.01, to compare the differences between the two indicated groups. AAN = aristolochic acid nephropathy; BW = body weight; PE = propolis extract.

Corresponding to the findings in Figure 1C, uremic mice with AAI treatment had a significant decrease in BW through the experimental period (Figure 2A). Compared with AAI-treated groups, mice not receiving AAI showed a steady increase in BW. Furthermore, an atrophic kidney due to fibrosis is characterized by shrinkage to an abnormal size and weight. TIF is a key feature and common outcome of inflammation across all kinds of advanced CKD [19]. Our data demonstrated that the size and weight in kidneys of AAN mice were lower than those without AAI treatment, indicative of renal atrophy. Although AAN group exhibited the smallest kidney size and mass than the other groups, PE treatment ameliorated such renal atrophy in AAN-PE group (Figure 2B,C). In the histopathological evaluation of H&E stain, renal tubular cells in AAN group exhibited the most prominent cytoplasmic vacuolation, loss of cell-cell adhesion, apical-basal polarity, thinning/attenuation of the epithelial layer lining tubules and irreversible cellular change with eventual cell loss and shedding into tubular lumina (Figure 2D). Vacuoles lacked uniformity of size or discrete outlines within tubules. Recent evidence unveils that cytoplasmic vacuolation is the necrotic nature of proximal tubular epithelial cells in response to renal injuries [20]. Remarkable tubular atrophy, glomerular obsolescence and shrunken, scarred kidneys in AAN models were compatible with hallmarks of ESKD in humans. Above findings of renal damages were in accordance with previous studies [21] and typical manifestations in advanced CKD [22]. Fibrosis and fibrous replacement are the eventual sequelae of CKD, regardless of cause. Although PE treatment reversed progressive TIF in advanced CKD, further investigations of the therapeutic effects on renal function and uremic burden were required.

### 2.3. PE Treatment Imporved Renal Function Indicators and Plasma Retention of Uremic Toxins (IS and pCS) in AAN Model 

**Figure 3:**
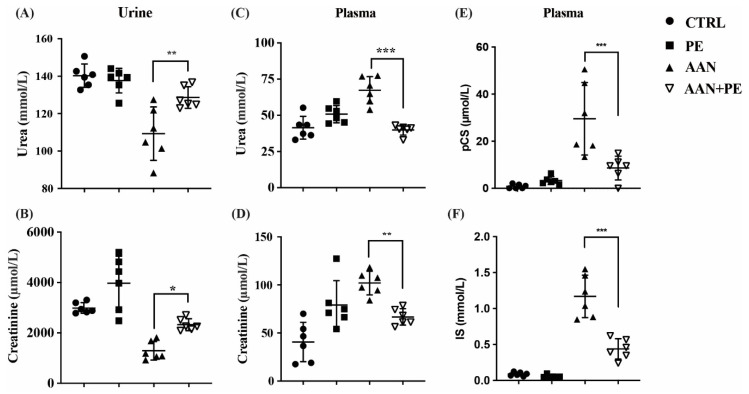
Comparisons of renal function indicators and plasma concentrations of uremic toxins (IS and pCS) among the control, PE, AAN and AAN-PE treatment groups. (**A**,**B**) The urine excretion capacity of UUN and creatinine in C57BL/6 mice with AAN were lowest, and PE treatment improved above renal function indicators. (**C**,**D**) AAN group without PE treatment exhibited the highest plasma concentration of BUN and creatinine, and PE treatment improved above renal function indicators. (**E**,**F**) AAN group without PE treatment exhibited the highest accumulation of IS and pCS in plasma, and PE treatment improved such retention of uremic solutes. AAN = aristolochic acid nephropathy; BUN = blood urea nitrogen; IS = indoxyl sulfate; pCS = p-cresyl sulfate; PE = propolis extract; UUN = urine urea nitrogen. *n* = 6 in each group; *** *p* < 0.001, ** *p* < 0.01 and * *p* < 0.05 to compare the differences between the two indicated groups.

As we had already proven that PE treatment reversed AAI-induced TIF and shrunken kidney, we aimed to investigate therapeutic effects of PE on renal functions and uremic burden. The urinary excretion capacity of UUN and Cr was lowest in the AAN group without PE treatment than the other groups, and PE treatment improved the urinary excretion of waste products in AAN-PE group (Figure 3A,B). AAN group without PE treatment also exhibited the highest plasma concentration of BUN and Cr, and PE treatment improved above renal function indicators (Figure 3C,D). Moreover, the AAN group without PE treatment exhibited the highest accumulation of IS and PCS in plasma, and PE treatment improved uremic burden in circulation (Figure 3E,F).

### 2.4. PE Treatment Attenuated Tissue Expressions of TIF, Fibrotic EMT and TGF-β Signaling Transduction Pathways

**Figure 4:**
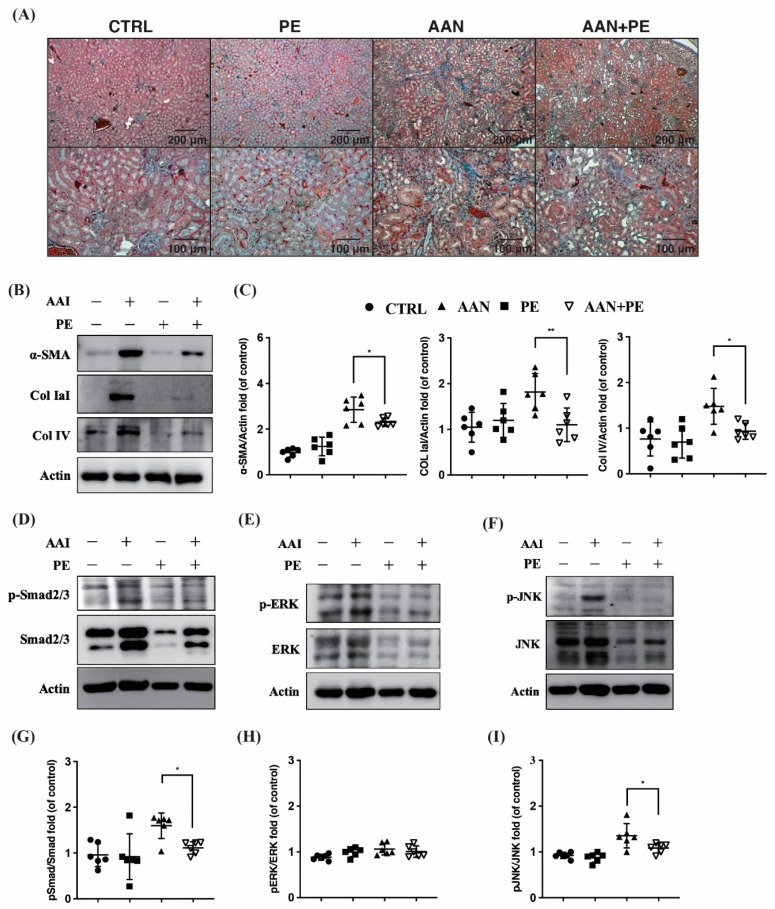
Tissue expressions of TIF, fibrotic EMT and TGF-β signaling transduction pathways among the control, PE, AAN and AAN-PE treatment groups. (**A**) C57BL/6 mice with AAN exhibited the most prominent TIF in Masson’s trichrome stain, and PE treatment ameliorated such renal injury. (**B**) PE treatment suppressed α-SMA expression and deposition of Col IaI and IV in the fibrotic EMT. (**C**–**I**) PE treatment attenuated not only SMAD 2/3-dependent pathways but also SMAD-independent JNK/ERK activation in the signaling cascades of TGF-β family. AAI = aristolochic acid I; AAN = aristolochic acid nephropathy; Col = collagen; TGF-β = transforming growth factor-β; PE = propolis extract; TIF = tubulointerstitial fibrosis. *n* = 6 in each group; ** *p* < 0.01 and * *p* < 0.05 to compare the differences between the two indicated groups.

Our previous research has reported non-specific ROS scavenger ameliorates TIF and uremic lung injury in CKD mouse models [8,16]. To investigate this furher, therapeutic effects of the potent antioxidant PE on AAI-induced TIF were evaluated here. Our results illustrated the most prominent TIF in Masson’s trichrome stain was found in AAN group than the other groups. As expected, PE treatment attenuated such renal injury, suggesting that the above fibrotic process was disrupted (Figure 4A). AAN group exhibited higher expressions of α-SMA, collagen IaI and IV, indicative of fibroblasts activation and ECM production. Indeed, PE treatment suppressed α-SMA expression and ECM deposition of collagen IaI and IV in the process of fibrotic EMT (Figure 4B). In addition, PE treatment t disrupted not only SMAD 2/3-dependent pathways but also SMAD-independent JNK/ERK activation in the signaling cascades of TGF-β family. (Figure 4C–E).

AA-I, a potent nephrotoxic agent, induces cytoplasmic vacuolation, cell necrosis and detachment in the tubular epithelium. To ameliorate such renal injury, SMAD 2/3-dependent and SMAD-independent JNK/ERK pathways are activated along with EMT-like phenotypic changes, leading to TIF and ECM deposition (Col IaI and IV). PCS/IS retention following tubular epithelial damages and impaired renal clearance inhibits renal metabolic capacity and induces further TIF. While PE therapy disrupts the vicious cycle through not only SMAD 2/3-dependent pathways but also SMAD-independent JNK/ERK activation in the signaling cascades of TGF-β family, facilitating renal excretion of PCS/ IS in AAN. In light of this, PE may be capable of developing a new potential drug to treat AA-induced toxic events and uremic toxin retention in CKD patients. AA-I = aristolochic acid I; AAN = aristolochic acid nephropathy; CKD = chronic kidney disease; Col = collagen; ECM = extracellular matrix; EMT = epithelial-mesenchymal transition; IS = indoxyl sulfate; PCS = *p*-cresyl sulfate; PE = propolis extract; SMA = smooth muscle actin; TGF-β = transforming growth factor-β; TIF = tubulointerstitial fibrosis.

## 3. Discussion

**Figure 5:**
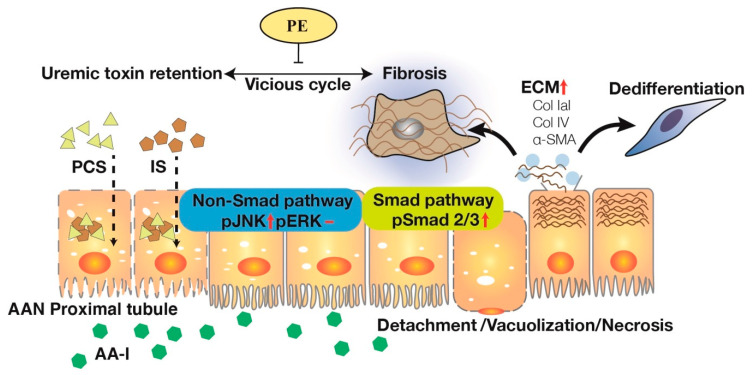
Illustrating potential therapeutic mechanisms of PE for AA-I induced CKD progression and uremic toxin retention.

CKD is a critical health concern and economic burden globally [2]. As a result of the current huge unmet need for new and reliable therapies, CKD prevalence is expected to increase. The reasons why CKD is so difficult to treat are intricate. Irreversibility of renal fibrosis, decline in glomerular filtration rate, and retention of uremic solutes reflect the trilogy of CKD progression. A progressive TIF due to various etiologies is an irreversible common process of CKD, ultimately resulting in renal atrophy, ESKD, uremic toxin-related morbidities and mortality [4]. Here, we provided a comprehensive treatment for AAI induced TIF and uremic solute retention through the investigation of AAN model. Conventionally, organ fibrosis has been considered potentially irreversible. However, compelling evidences from animal models and human studies have demonstrated that if the injury is removed, early stages of organ fibrosis may be reversible [23]. There is a clear need for safe and effective therapeutic regimens focusing on TIF to timely preserve renal function in patients with AAs intoxication, preventing from ESKD and fatal urothelial cancer in clinical medicine. PE, a potent natural antioxidant with multi-components, serves as a multi-targeted regimen in inflammation [12], diabetes [13], and cancers [15]. Our recent study unraveled that the scavenging of ROS by antioxidants ameliorates obesity-related kidney fibrosis [16]. Considering pro-oxidant and pro-fibrotic effects of AAs and uremic toxins on human organ systems, PE could be a potential breakthrough treatment for AAs toxicity and CKD progression. Through testing the therapeutic effects of PE on TIF and PCS/IS retention in our AAN model, major breakthroughs were achieved and novel findings markedly advance our understanding of irreversible process in CKD (Figure 5). Several important findings in this work deserve a more in-depth discussion.

To date, AAN remains regularly reported all over the world [24]. The incidence of AAN is probably highly underestimated due to the presence of AAs in traditional herbal remedies worldwide and the poor awareness of the disease. The majority of AAN patients presented BW loss, anemia, mild proteinuria, hypertension, a rapid decline of renal function and reached ESKD at the end of the seven-year follow up [25]. The progression of CKD can continue despite removing exposure to AAI [21]. Furthermore, AAI also suppressed expression of epidermal growth factor in tubular epithelial cells, indicating lack of tubular regeneration [21]. Concerning this critical issue, typical manifestations of AAI induced progressive CKD are increasingly recognized through both human and animal models. Macroscopically, intoxicated kidneys were shrunken, asymmetrical and irregular in cortical outline. Biopsies of human cases revealed interstitial inflammatory infiltrate and evidence of tubular necrosis. Microscopically, kidneys with AAN depicted extensive paucicellular interstitial fibrosis with atrophy and loss of tubules initiating from the peripheral cortex and progressing towards the deep cortex [26,27]. Progressive renal fibrosis featured with prominent tissue scarring, glomerulosclerosis, tubular atrophy and TIF is considered as a final common process of CKD leading to ESKD. Irrespective of the nature of the initial renal injury, the degree of TIF correlates well with the decline of the renal function and long-term prognosis. To mimic typical findings of TIF in humans with advanced CKD, we developed an experimental mouse model of AAN. Our data pointed out that plasma Cr was positively correlated with BUN (*r* = 0.59; *p* < 0.05), PCS (*r* = 0.51; *p* < 0.05) and IS (*r* = 0.55; *p* < 0.05), and negatively correlated with UCr (*r* = −0.51; *p* < 0.05) and UUN (*r* = −0.61; *p* < 0.01) (Figure 1B). The above results demonstrated that AAN induced renal function decline led to the accumulation of various uremic toxins in the circulation and impaired urinary excretion of waste products (Figure 1). It is a vicious cycle that uremic retention solutes following impaired renal clearance could exhibit pro-oxidant [28], pro-inflammation [29] and pro-fibrotic effects [30] on renal tubulointerstitium, leading to progressive TIF [31]. As expected, mice in AAN group exerted the most prominent TIF and the highest expressions of α-SMA, collagen IaI/IV, SMAD 2/3 and JNK/ERK than the other groups (Figure 4). Moreover, BW of AAN mice is negatively correlated with BUN (*r* = −0.71; *p* < 0.01), Cr (*r* = −0.52; *p* < 0.05), PCS (*r* = −0.78; *p* < 0.01) and IS (*r* = −0.84; *p* < 0.01), respectively (Figure 1C). Our findings imply that cachexia is present in the uremic milieu, corresponding with previous clinical observation and basic research [17,18].

Molecular mechanisms involved in TGF-β overexpression remain to be fully elucidated. Pozdzik et al. reported that vimentin and α-SMA-positive cells accumulated in the renal interstitium, along with an overexpression of TGF-β in rats with AAN [32]. TGF-β1 upregulation has emerged as the key driver of matrix synthesis, inhibition of matrix degradation and stimulator of myofibroblast activation [33,34]. It has become more evident that profound reduction of peritubular capillaries in AAN resulting in hypoxia and tubular cell death, resulting in further progression to fibrogenesis [1]. TGF-β1 is a multi-functional cytokine that plays a fundamental role in regulating inflammation, cellular behaviors, fibrotic EMT, fibroblast viability and collagen degradation [35,36]. Smad2 and Smad3 serve as the two major downstream regulators that promote TGF-β1-mediated tissue fibrosis [37]. Deletion of Smad3 has been demonstrated to protect against several kidney disease, including AAN [38]. It is now well accepted that TGF-β/Smad signaling is the major pathway for renal fibrogenesis. Nevertheless, TGF-β1 can induce renal fibrosis in both canonical (SMAD-dependent) and non-canonical (SMAD-independent) signaling pathways, resulting in activation of myofibroblasts, excessive production of ECM and inhibition of ECM degradation [39,40]. TGF-β1-mediated non-SMAD pathways were involved in various branches of mitogen-activated protein kinase (MAPK) pathways, including the JNK and ERK signaling cascades [41]. In response to TGF-β1, the co-operation between SMAD and non-SMAD signaling pathways determines cell fate of fibrotic EMT. In our study, PE treatment did ameliorate TIF in Masson’s trichrome stained tissue (Figure 4A). In addition, PE treatment suppressed α-SMA expression and deposition of collagen IaI and IV in the process of fibrotic EMT (Figure 4B). Furthermore, PE treatment attenuated not only SMAD 2/3-dependent pathways, but also SMAD-independent JNK/ERK activation in the signaling cascades of TGF-β family (Figure 4C–E). In light of this, PE is considered to be a multi-targeted agent that exerts therapeutic effects on progressive CKD.

Our study has several limitations. To begin with, the amount of food intake and metabolic efficiency were not measured. Next, further parameters were not evaluated for renal function decline, e.g., proteinuria, cystatin C, β-trace protein, inulin and iohexol. In addition, expressions of picrosirius red staining and TGF-Beta proteins in the kidneys were not determined in our AAN models.

In conclusion, our research elucidates AAI damages tubular epithelium by TGF-β family signaling pathways and fibrotic EMT, leading to TIF, ECM deposition and PCS/IS retention. Notably, the natural product PE exerts nephroprotetive effects through the regression of renal fibrosis, thereby improving renal clearance of uremic toxins. Furthermore, PE treatment attenuates AA toxicity through disrupting not only SMAD 2/3-dependent pathways but also SMAD-independent JNK/ERK activation in the intricate cascades of TGF-β family. In light of multi-faced toxicity of AAs, PE may be capable of developing a new potential drug to treat CKD patients exposed to AAs in the future.

## 4. Materials and Methods

### 4.1. Creating Animal Models to Mimic TIF in Humans with Progressive CKD

To mimic TIF in humans with progressive CKD, we developed an AAN model using seven-week-old C57BL/6 mice by intraperitoneal (IP) injection of aristolochic acid I (AAI, Sigma Aldrich, Wuxi, China) 3 mg/kg once every 3 days for 6 weeks. The details of the study protocol and materials for AAN were also mentioned previously [8]. All animals were randomly divided into four groups of six mice each: vehicle-treated control group (normal renal function), PE-treated group (normal renal function), AAN group and PE-treated AAN group. PE was collected in Taiwan using propolis collectors as described previously [12]. The dosage and experimental administration were presented here: Group I (control; IP injection of vehicle (DMSO, Sigma Aldrich, China) once every 3 days for 6 weeks and orally administered with vehicle (distilled water, 200 μL) everyday, 12 weeks; *n* = 6). Group II (PE alone; IP injection of vehicle and orally administered with PE (0.2 mg/kg in 200 μL vehicle), 12 weeks; *n* = 6). Group III (AAI treatment; IP injection of AAI and orally administered with vehicle (200 μL) everyday, 12 weeks; *n* = 6) and Group IV (PE + AAI treatment; IP injection of AAI and orally administered with PE, 12 weeks; *n* = 6). All mice were maintained at temperature (23 ± 3 °C) and relative humidity (40–60%) on a 12 h light/dark cycle and allowed free access to standard rodent chow and tap water. Animal experiments were handled according to the guidelines of the Institutional Animal Care and Use Committee of the National Ilan University (approval number: 107-17; date: 16 December 2018) and NIH Guides for the Care and Use of Laboratory Mouse body weight was measured at least once a week throughout the 12 weeks. Kidneys were removed at termination and directly compared.

### 4.2. Tissue Preparation for Histopathological Evaluation of H&E Stain

Mice were anaesthetized via the inhalation of isoflurane and euthanized by cervical dislocation. Kidneys were removed and fixed in 10% formalin. Specimens were embedded in paraffin and sliced into 2–3 μm in thickness. Subsequently, the kidney tissues were stained with Hematoxylin-eosin (H&E stain). The images were captured using a Nikon Digital Camera Microscope (Nikon, Tokyo, Japan).

### 4.3. Masson’s Trichrome Staining Method

Masson’s trichrome staining method was used to determine the extent of collagen deposition and fibrosis in mouse kidney tissues. In the corresponding area, H&E staining of the adjacent paraffin section was performed for comparisons of tissue morphology. The experiments were conducted as follows: sections were first deparaffinized and rehydrated in ethanol/water solutions then post-fixed with Bouin’s solution for 1 h at room temperature. The fixation buffer was removed, and slides were stained with iron hematoxylin, Biebrich scarlet-acid fuchsin, and phosphomolybdic-phosphotungstic acid sequentially for 10 min per stain. Slides were then stained with Aniline blue. Finally, slides were washed in 10% acetate solution for 3–5 min and mounted in the mounting medium for observations to be made. The slices were visualized by an Olympus BX-41 microscope.

### 4.4. Biochemical Assays of Urea nitrogen, Creatinine (Cr), PCS and IS

Plasma and urine concentrations of urea nitrogen and Cr were determined by MeDiPro CREA (BC-0017) and MeDiPro BUN (BC-0012). PCS and IS in plasma samples were analyzed by a Mass Spectrometer Analytical System (Thermo Fisher Scientific Inc., Waltham, MA, USA) and UHPLC analytical system (Thermo Fisher Scientific Inc., Waltham, MA, USA). The Xcalibur software (version 2.2, Thermo-Finnigan Inc., San Jose, CA, USA) was used for method setup and data processing.

### 4.5. Western Blot Analysis

The kidney tissues were homogenized in radioimmunoprecipitation assay buffer (Millipore # 20-188) and incubated at 4 °C for 30 min. The supernatant was collected after centrifugation for 20 min at 14,000 rpm, 4 °C. Protein concentration was assessed by a Bradford protein assay kit (Bio-Rad, Hercules, CA, USA). Sixty micrograms of protein were electrophoresed on SDS/PAGE gels. The gels were transferred to a PVDF membrane (Millipore, #IPVH00010) and incubated in 1% skim milk blocking buffer overnight at 4 °C. The antibodies used included anti-α-smooth muscle actin (SMA) (GeneTex, GTX100034, 1:1000, 4 °C, o/n), Col IaI (OriGene, TA309097, 1:1000, 4 °C, o/n), Col IV (Abcam, ab6586, 1:1000, 4 °C, o/n), p-Smad2/3 (Cell Signaling, 8828S, 1:1000, 4 °C, o/n), Smad2/3 (Cell Signaling, 5678S, 1:1000, 4 °C, o/n), p-JNK (Cell Signaling, 9255s, 1:1000, 4 °C, o/n), JNK (Cell Signaling, 9252, 1:1000, 4 °C, o/n), p-ERK1/2 (Cell Signaling, 9106S, 1:1000, 4 °C, o/n), ERK1/2 (Signaling, 9102, 1:1000, 4 °C, o/n) and β-actin (Santa Cruz, sc-47778, 1:2000, 4 °C, o/n). Appropriate horseradish peroxidase-conjugated secondary antibody was incubated for 60 min at RT. Positive immunostaining was detected with the Super Signal West Pico enhanced chemiluminescence substrate (Thermo Fisher Scientific, Hudson, NH, USA). Images were digitally acquired by LAS-3000 Plus instrument (Fuji Film, Tokyo, Japan). The reactions were quantified and normalized to the β-actin control band using ImageJ 1.48q (National Institutes of Health, Bethesda, MD, USA).

### 4.6. Statistical Analysis of Data

All data are expressed as the mean ± SEM using the GraphPad Prism Program (GraphPad, San Diego, CA, USA) or SPSS version 22.0 (IBM, Armonk, NY, USA). Spearman’s test with linear regression analysis was used for correlation data. Quantitative data were analyzed with Kruskal and Wallis test. A *p*-value < 0.05 was considered statistically significant for each of the experiments.
